# HIIT and MICT attenuate high-fat diet-induced hepatic lipid accumulation and ER stress via the PERK-ATF4-CHOP signaling pathway

**DOI:** 10.1007/s13105-022-00884-7

**Published:** 2022-03-22

**Authors:** Zhang Yuan, Liu Xiao-wei, Wei Juan, Liu Xiu-juan, Zhang Nian-yun, Sheng Lei

**Affiliations:** 1 The Key Laboratory of Systems Biomedicine, Ministry of Education, and The Exercise Translational Medicine Centre, Shanghai Center for Systems Biomedicine, Shanghai Jiao Tong University, Shanghai, 200240 China; 2 School of Sports and Health, Nanjing Sport Institute, Nanjing, 210014 China; 3 Jiangsu Collaborative Innovation Center for Sport and Health Project, Nanjing, 210014 China; 4 Huishan District Rehabilitation Hospital, Wuxi, 214100 China

**Keywords:** Obesity, High-intensity interval training, Moderate-intensity continuous training, Hepatic lipid accumulation, ER stress

## Abstract

Fatty liver can be induced by dietary habits and lifestyle and is directly related to obesity. Although the benefits of exercise interventions for reduction of liver fat have recently been acknowledged, the underlying mechanisms remain unclear. Thus, our present study investigated the effects of high-intensity interval training (HIIT) and moderate-intensity continuous training (MICT) on high-fat diet-induced hepatic lipid accumulation, and explored the role of endoplasmic reticulum (ER) stress signaling pathways. To establish an obesity model, rats were fed with a normal standard diet or a high-fat diet (45% kcal as fat). Then, both lean and obese rats were divided into three subgroups: sedentary control (LC, OC) groups, high-intensity interval training (LHI, OHI) groups, and moderated-intensity continuous training (LMI, OMI) groups (*n* = 10). Rats in the exercise group underwent a swimming training protocol for 8 weeks. After the experimental period, serum and liver tissues from different groups were dissected for morphological and biochemical analyses. The results showed that with HIIT and MICT interventions, body weight and serum inflammatory markers (e.g., MCP-1, IL-1β, and TNF-α) were reduced in obese rats. Interestingly, HIIT was more effective in ameliorating liver triglyceride content and enhancing mitochondrial metabolic-enzymatic activity than was MICT in obese rats. Both HIIT and MICT conferred beneficial properties through upregulating Nrf2 expression, improving antioxidant enzyme activities and reduction of hepatic ER stress, which may have been regulated by the Bip-mediated PERK-ATF4-CHOP pathway. In conclusion, our findings confirmed the effectiveness of HIIT and MICT, particularly HIIT, in mitigating hepatic lipid accumulation.

## Introduction

Obesity is a global health problem, and it is the most important risk factor for fatty liver development [20]. People who suffer from obesity are much more likely to develop a fatty liver and have it progress to more dangerous conditions, such as nonalcoholic steatohepatitis (NASH), cirrhosis, and liver cancer [16]. A prospective study from Vilar-Gomez et al. reported that the highest rates of NASH resolution and fibrosis regression occurred in patients with weight losses >10% [30]. Therefore, obesity is characterized not only by excessive fat deposition but also by important complications such as nonalcoholic liver steatosis.

Through well-tuned coordination with adipose, muscle, and other tissues, the liver plays an essential role in the maintenance of lipid homeostasis and energy balance. Hepatic lipid accumulation can result from excessive lipid influx or impaired lipid efflux, while β-oxidation and lipoprotein secretion decrease hepatic lipid content. However, unhealthy lifestyles of nutritional overload and physical inactivity may tilt the balance of lipid homeostasis and disrupt one’s body sensitivity to insulin. Following over-nutrition and obesity, hepatic fatty acid metabolism is altered, commonly leading to the accumulation of triglycerides within hepatocytes, and to a clinical condition known as nonalcoholic fatty liver disease (NAFLD). In general, caloric restriction is the most important dietary intervention and has the biggest impact on reducing weight and improving liver conditions [2]. Increased physical activities have also been shown to reduce hepatic steatosis, visceral adipose tissue, and plasma free fatty acids, decreasing the likelihood of developing NAFLD and NASH [5, 10, 12].

Hepatocytes are responsible for lipogenesis and cholesterol biosynthesis, as well as glucose and xenobiotic metabolism. To fulfill their myriad of metabolic functions, hepatocytes are enriched in both smooth and rough endoplasmic reticulum (ER). The ER is not only important for protein synthesis and folding but also crucial for lipid synthesis and metabolism. Abnormal lipid accumulation often coincides with insulin resistance in steatotic livers, and it is associated with oxidant stress and perturbed ER proteostasis in hepatocytes [26]. Various components of ER stress play roles in regulation of lipid metabolism, and the PERK-eIF2α-ATF4 pathway has been identified as a critical component required for regulating lipogenesis and hepatic steatosis [1].

Moderated-intensity continuous training (MICT) is a traditional method for weight control. In recent times, high-intensity interval training (HIIT) has become a popular alternative, primarily because of its time efficiency. Numerous studies have demonstrated that exercise training improves systemic markers of liver function and intrahepatic fat in mild-to-advanced NAFLD [5, 10, 12]. Data from an animal study showed that knee loading significantly decreased the histological severity of hepatic steatosis and downregulated biomarkers related to ER stress (e.g., GRP78, p-eIF2α, and ATF4) compared to those of obese mice [23]. In addition, da Luz et al. indicated that the increased rat hepatic protein expression levels of p-PERK and eIF2α in high-fat diet (HFD)-induced obesity were mitigated after a swimming training program [6]. However, another study also showed that exercise training per se was not able to modulate the hepatic expression levels of CHOP or ATF-4 in mice subjected to a HFD [8]. Hence, even though physical exercise has been suggested as a protective stimulus against ER stress–induced abnormal lipid metabolism in the liver, it is not clear whether these effects occur following all exercise types (e.g., swimming vs treadmill, voluntary physical activity vs endurance training, and duration vs intensity workouts).

The present study aimed to investigate the effects of two exercise protocols, HIIT and MICT, on hepatic lipid accumulation and the role of ER stress signaling pathways in HFD-induced obese rats.

## Material and methods

### Animals and experimental design

**Fig. 1:**
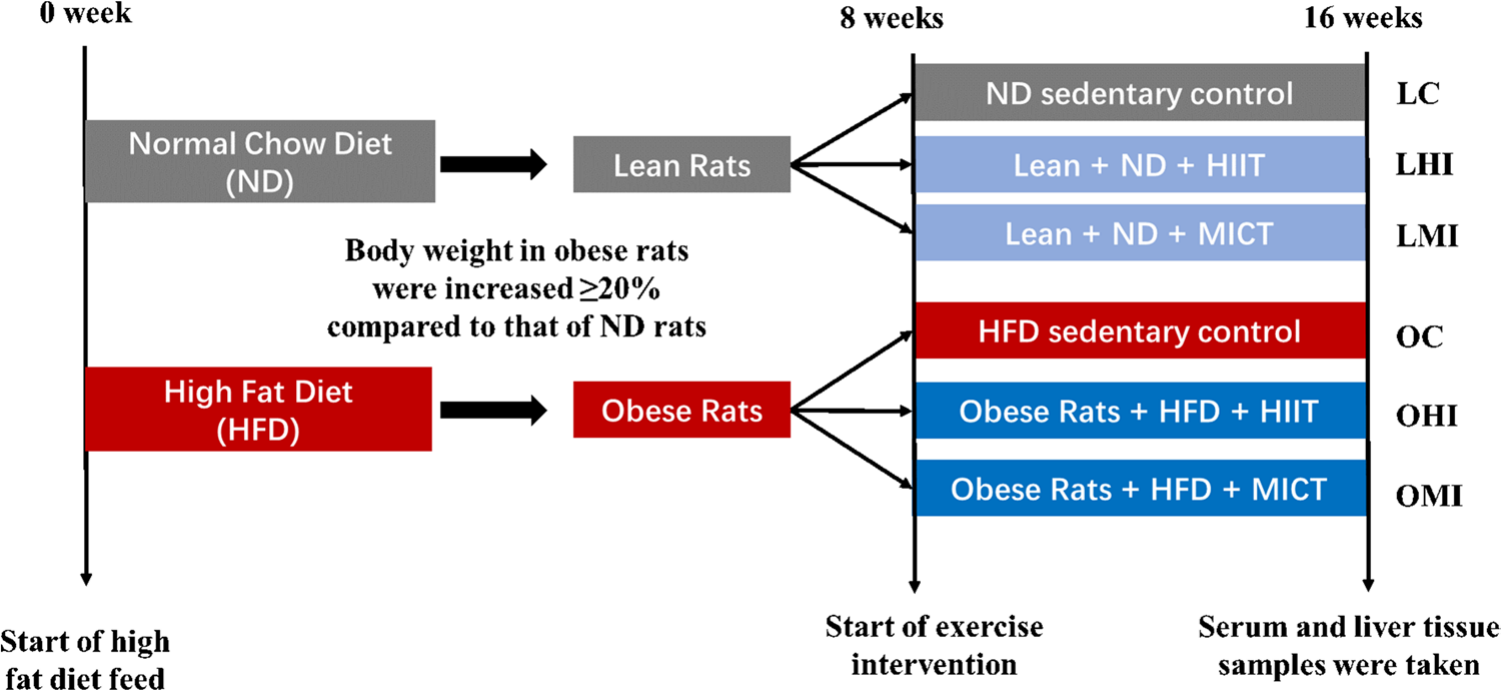
Overall design of this study. After 1 week of acclimatization, mice were fed a normal chow diet (ND group) or high-fat diet (HFD group) for 8 weeks. After 8 weeks of diet, obese rats were defined as those with at least a 20% increase in body weight compared to that of ND diet rats. Lean and obese rats were randomly submitted to the sedentary control group and HIIT or MICT exercise intervention protocol for 8 weeks, which were randomly assigned to six groups: lean-feeding ND-sedentary (LC group, *n* = 10), lean-feeding ND-HIIT (LHI group, *n* = 10), lean-feeding ND-MICT (LMI group, *n* = 10), obese-feeding HFD-sedentary (OC group, *n* = 10), obese-feeding HFD-HIIT (OHI group, *n* = 10), obese-feeding HFD-MICT (OMI group, *n* = 10). Serum samples and liver tissue were obtained at 16 weeks

Sixty male Sprague-Dawley rats (age, 4 weeks; weight, 68.4 ± 2.4 g at the beginning of the experiment) were purchased from Qinglongshan Animal Breeding Farm (Nanjing, China). All of the rats were reared in a laboratory (temperature 24–25°C, humidity 70–75%, with a 12 h light/dark lighting regimen) and were fed either a standard diet or a HFD (45% kcal as fat, Research Diets: D12451) of pellets, and water was provided ad libitum. Rats were fed with a normal standard diet or a HFD for 8 weeks without exercise regimens. Body weight was monitored once weekly during the study. After these periods, obese rats were defined as those with at least a 20% increase in body weight compared to that of standard-diet rats. Following confirmation of obesity, lean and obese rats were each divided into three subgroups, as follows: (1) lean sedentary control group (LC); (2) lean high-intensity interval training group (LHI); (3) lean moderated intensity continuous training group (LMI); (4) obese sedentary control group (OC); (5) obese high-intensity interval training group (OHI); and (6) obese moderated intensity continuous training group (OMI) (*n* = 10) (Fig. 1). All of the animal procedures were performed in accordance with the “Guide for the Care and Use of Laboratory Animals” (National Institutes of Health, the USA) and all of the experiments were received and approved by the Ethical Committee of Animal Experiments, Faculty of Nanjing Sport Institute, Nanjing, China (Protocol No. 2019-010).

### Training protocols

Swimming training sessions were performed in an individual glass chamber (60-cm high × 30-cm diameter) with water temperature controlled at 31 ± 1°C. The swimming training was conducted during 8 weeks, with a weekly frequency of 5 days (Monday to Friday) during the morning (∼8:00 AM and 11:00 AM). The load intensity was attached to the rat’s tail and was individually adjusted in each exercise session according to the rat’s body mass. The progressions of load intensity, volume of interval, and continuous training were adapted from protocols describe by Terada et al. [25]. Regarding load intensity, a previous study indicated that lactate threshold was achieved with loads between 5 and 6% of a rat’s body mass [11]. Therefore, the continuous protocol used in the present study consisted of low/moderate intensity (load between 0 and 3% of body mass), while the interval protocol that we used consisted of high intensity (load between 5 and 16% of body mass). Table 1 shows the swimming training protocols used in the present study. After the exercise sessions, rats were dried and returned to the bioterium standard conditions.

**Table 1:** High-intensity interval training and moderate-intensity continuous training

High-intensity interval training					Moderate-intensity continuous training			
Week	Set	Time	Rest	Load (%body weight)	Week	Set	Time	Load (%body weight)
1	5	1min	1min	0–5%	1	1	30min	0%
2	5	1min	1min	7%	2	1	40min	0%
3	5	1min	1min	8%	3	1	30min	1%
4	5	1min	1min	10%	4	1	40min	1%
5	14	20s	10s	13%	5	1	40min	2%
6	14	20s	10s	14%	6	1	50min	2%
7	14	20s	10s	15%	7	1	50min	3%
8	14	20s	10s	16%	8	1	60min	3%

### Sample collection

Serum and liver tissue samples were taken at least 24 h after the last training sessions. Rats were deprived of food for 12 h and were then sacrificed. Blood samples were collected from the retrobulbar vein, followed by centrifugation at 3,000 rpm for 10 min to separate the serum, which was stored at −80°C until further use. The liver samples were dissected and rapidly weighed. Portions of the liver were fixed immediately in 10% formalin for future histological observation and the remaining portions were stored at −80°C until further use.

### Histological examinations

Liver tissues were harvested, cut into small pieces, fixed in 4% paraformaldehyde, and embedded in pre-cooled optimal cutting compound (OCT) for cryostat sectioning. The OCT-embedded samples were serially sectioned at 6μm and stained with Oil Red-O to visualize accumulation of hepatic lipid droplets. The samples were observed and imaged under a microscope (IX3-AN, Olympus, Japan). To estimate adipogenesis of the liver, after removing the staining solution, Oil Red-O was extracted by isopropanol and its optical density was monitored spectrophotometrically at 492 nm via a microplate reader for quantitative analysis with Image Pro Plus software.

### Biochemical analysis

Serum total cholesterol (TC), triglyceride (TG), high-density lipoprotein cholesterol (HDL), FFA and liver reactive oxygen species (ROS) level, superoxide dismutase (SOD), glutathione peroxidase (GSH-Px), and catalase (CAT) activity were measured via a microplate assay (Tecan 2000, Japan) from Nanjing Jiancheng Bioengineering Institute (China). Serum MCP-1, IL-β, and TNF-α assays were conducted according to the manufacturer’s protocols using a Bio-Plex Pro Rat Adipokine Magnetic Bead Panel (RADPKMAG-80K, 3017598) with a Luminex 200 system (Austin, TX, USA) from Wayen Biotechnologies Shanghai, Inc. Liver TG was extracted with chloroform/methanol (2:1), evaporated, and then dissolved in ethanol. TG content was determined with an enzyme reaction kit (Nanjing Jiancheng Bioengineering Institute, China). TG levels were normalized to the protein concentration of each sample.

### Enzyme-linked immunosorbent assays (ELISAs)

The activities of hepatic enzymes—including CPT-1α, β-hydroxyacyl-CoA dehydrogenase (β-HAD), ACC, and citrate synthase (CS)—were measured using enzyme-linked immunosorbent assay (ELISA) kits (Shanghai Enzyme-linked Biotechnology Co., Ltd.). All of the measurements were carried out according to the manufacturers’ instructions.

### Western blotting analyses

Protein contents were extracted either from nuclear (Thermo Fisher Scientific, 78833, USA) or whole muscle lysates from liver tissues. Whole muscle protein was isolated by lysing in RIPA lysis buffer (Beyotime, P0013B, China) supplemented with phosphatase and protease inhibitor cocktails (Beyotime, P1048), after which the supernatant was collected. The concentrations of nuclear and whole muscle proteins in the acquired supernatants were determined via a Bradford assay kit (Thermo Fisher Scientific, 23227). Subsequently, an equal amount of protein was separated on a 10% polyacrylamide gel under reduced conditions and was then transferred onto a polyvinylidene difluoride (PVDF) membrane (Millipore, IPVH00010, USA). Proteins were separated by SDS-PAGE using a 7.5% (PGC-1α, phosphorylated-protein kinase-like endoplasmic reticulum kinase, P-PERK), (Histone, H3)) or 12.5% (ATF-4, (the C/EBP homologous protein, CHOP), (Binding immunoglobulin protein, Bip), GAPDH) polyacrylamide gel, and proteins were subsequently transferred onto a PVDF membrane. For the detection of proteins, commercially available antibodies were used for P-PERK (1:1000, 3179, Cell Signaling), H3 (1:10000, Ruiying Biological, RLM3038, China), Nrf2 (Proteintech, 16396-1-AP), Bip (1:800, Bioworld, BS6479), ATF4 (1:1000, Bioworld, BS1026, USA), CHOP (1:800, Bioworld, BS1136), PGC-1α (1:1000, Proteintech, 66369-1-Ig, China), and GAPDH (1:20000, Proteintech, 60004-1-Ig). Proteins were detected via chemiluminescence by using a High-sig ECL Western Blotting Substrate (Tanon, 180-501, China). Blots were quantified using a GelDoc Go Gel Imaging System and Image Lab software (BIO-RAD, USA).

### Statistical analyses

Values are given as the mean ± standard deviation (SD) for all variables. Data are expressed as the means ± SD and were analyzed using GraphPad Prism software, version 6.0 (GraphPad Software Inc., San Diego, CA). *t*-test, two-way analysis of variance (ANOVA), or one-way ANOVA was used when appropriate. Differences were considered to be statistically significant at *p* < 0.05.

## Results

### HIIT and MICT mitigate HFD-induced increases in body weight, liver weight, and adipose tissue weight

**Fig. 2:**
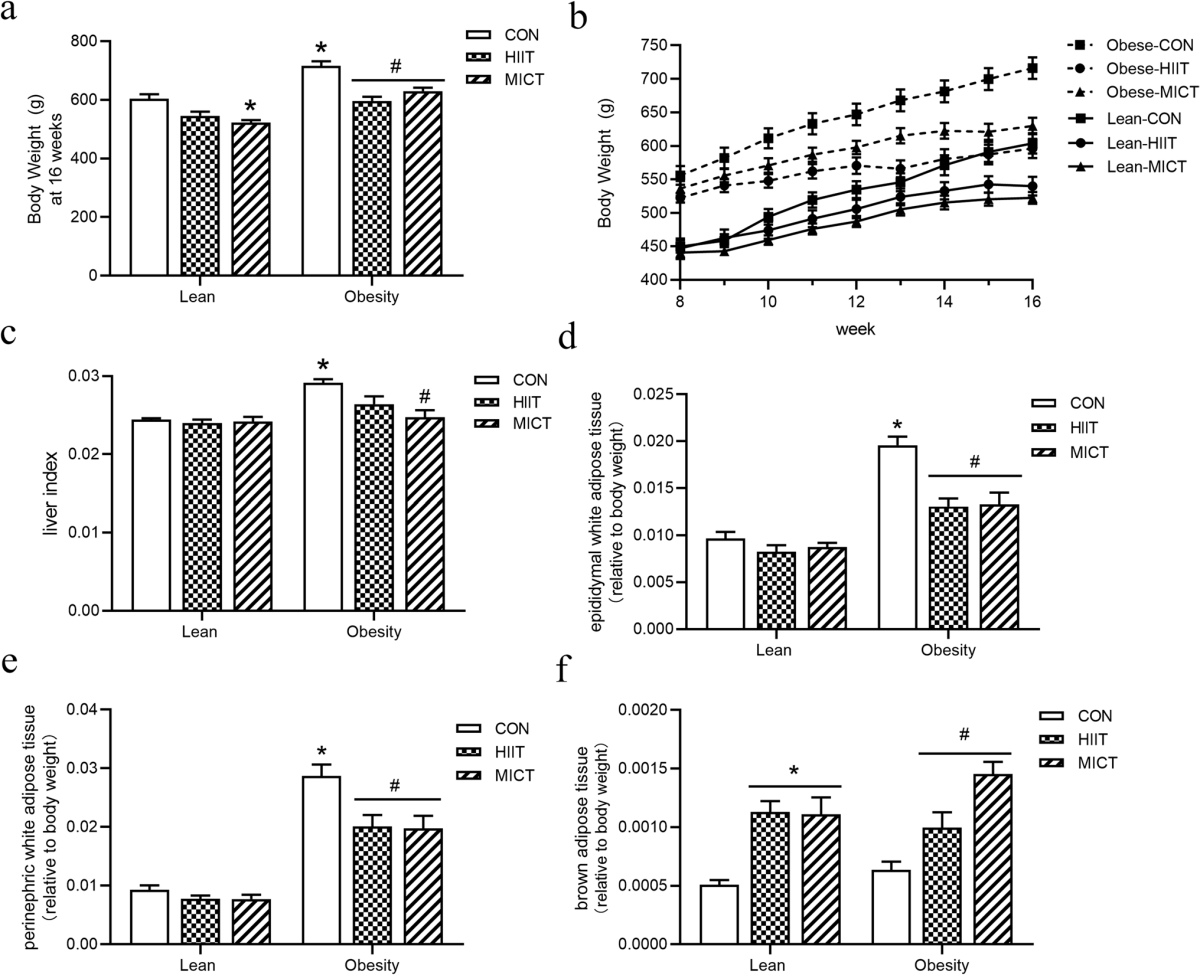
Effects of HIIT and MICT on body weight at the last week (**a**) and body weights from week 1 to week 8 (**b**). Changes in liver weight relative to body weight (**c**). Different adipose tissues relative to body weight: epididymal adipose tissue (eWAT) (**d**), perigonadal adipose tissue (pWAT) (**e**), and brown adipose tissue (BAT) (**f**). LC, lean + quiet control group; LHI, lean + high-intensity interval exercise training group; LMI, lean + moderated intensity continuous training group; OC, obesity + quiet control group; OHI, obesity + high-intensity interval exercise training group; OMI, obesity + moderated intensity continuous training group. Values are presented as the mean ± SD (*n* = 6–10 per group). *P < 0.05 vs. LC group (*t*-test or one-way ANOVA), #*P* < 0.05 vs OC group (one-way ANOVA)

An 8-week HFD significantly increased body weight gain and the weight of each white adipose tissue (WAT) deposit in obese rats compared to those of standard-diet-fed counterparts. HIIT and MICT decreased body weight in both diet types; HIIT had a significant effect on weight loss in obese rats, whereas MICT had more of an influence on weight loss in lean rats. In addition, both types of exercise interventions significantly decreased liver weight, epididymal white adipose tissue (eWAT) weight, and perigonadal white adipose tissue (pWAT) weight in HFD-fed groups compared to those of sedentary HFD-fed counterparts. However, the BAT percentage was significantly increased in the trained groups compared to that of untrained rats with either diet regimen (Fig. 2).

### Effects of HIIT and MICT on serum lipid levels and inflammatory markers

Both types of exercise training showed no significant effects on serum TG or Ch levels in the lean group, whereas a significant increase was observed for serum HDL-C after 8-week HIIT or MICT (Table 2). In terms of the obesity groups, HFD induced a remarkable increase in serum lipid levels when compared with those of the LC group. There was a trend toward a decrease in serum TG and HDL-C levels in OHI rats compared to those of OC rats, although this difference did not reach statistical significance. Moreover, MICT induced a marked reduction in serum TG, Ch, and HDL-C levels.

**Table 2:** Changes in serum lipid and inflammatory markers in rats of each group

	LC	LHI	LMI	OC	OHI	OMI
TG (mmol/L)	0.34±0.04	0.25±0.02	0.25±0.03	0.50±0.06*	0.40±0.06	0.31±0.05#
Ch (mmol/L)	1.10±0.14	1.06±0.09	0.93±0.08	1.64±0.15*	1.29±0.12#	1.09±0.08##
HDL-C (mmol/L)	0.21±0.02	0.27±0.02*	0.26±0.04*	0.35±0.04*	0.29±0.05	0.21±0.03#
MCP-1 (pg/ml)	185.44±24.44	204.13±18.32	219.62±14.15	420.28±51.18**	245.48±59.98##	182.45±22.51##
TNF-α (pg/ml)	0.86±0.04	0.95±0.21	1.16±0.17	2.65±0.42**	1.19±0.27##	0.91±0.11##
IL-1β (pg/ml)	53.40±33.19	32.70±8.82	30.12±9.18	139.49±16.82**	84.67±16.72#	80.45±23.19#

LC, lean + quiet control group; LHI, lean + high-intensity interval exercise training group; LMI, lean + moderated intensity continuous training group; OC, obesity + quiet control group; OHI, obesity + high-intensity interval exercise training group; OMI, obesity + moderated intensity continuous training group; TG, triglycerides; Ch, cholesterin; LDL-C, low-density lipoprotein cholesterin; MCP-1, monocyte chemotactic protein 1; IL-1β, interleukin-1 β; TNF-α, tumor necrosis factor alpha. Values are presented as the mean ± SD (*n* = 6–10 per group). *P < 0.05 vs LC group, **P < 0.01 vs LC group (*t*-test or one-way ANOVA), #*P* < 0.05 vs. OC group, ##*P* < 0.01 vs OC group (one-way ANOVA)

To further explore neuroinflammatory responses to diet and exercise interventions, we next evaluated serum inflammatory markers. As shown in Table 2, MCP-1, TNF-α, and IL-1β were markedly increased in the serum of the OC rats, whereas 8-week HIIT and MICT significantly ameliorated HFD-induced upregulation of these inflammatory factors.

### HIIT and MICT ameliorate HFD-induced hepatic lipid accumulation

**Fig. 3:**
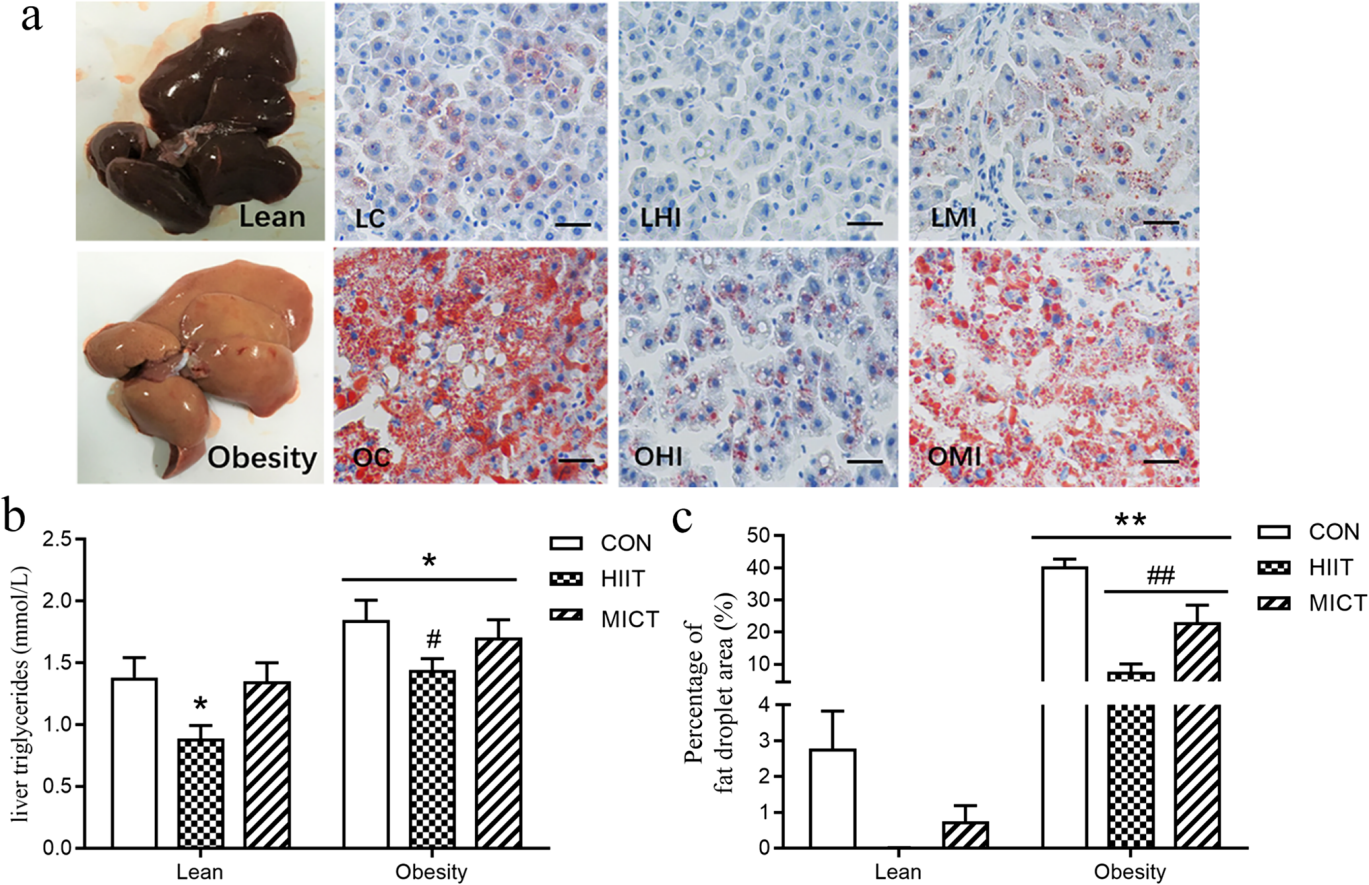
Effects of HIIT and MICT on liver lipid contents. Oil Red-O staining of liver tissue (**a**). Liver TG levels (**b**). Percentage of fat droplet area (%) (**c**). LC, lean + quiet control group; LHI, lean + high-intensity interval exercise training group; LMI, lean + moderated intensity continuous training group; OC, obesity + quiet control group; OHI, obesity + high-intensity interval exercise training group; OMI, obesity + moderated intensity continuous training group; values are presented as the mean ± SD (*n* = 6–10 per group). *P < 0.05 vs LC group (one or two-way ANOVA), **P < 0.01 vs LC group (two-way ANOVA), #*P* < 0.05 vs OC group, ##*P* < 0.01 vs OC group (one-way ANOVA)

TG levels were measured in liver lysates from lean and obese mice. Liver TG levels were significantly increased in obesity control mice compared with those in lean control mice, whereas liver TG levels were markedly reduced after 8-week HIIT. However, these levels did not change in the MICT group in both lean and obesity mice. Consistently, a significant increase in the accumulation of lipid droplets was observed in OC mice compared with that in LC mice, as determined by Oil Red-O staining (Fig. 3). In OHI and OMI groups, the accumulation of lipid droplets in hepatocytes was clearly reduced in exercised HFD groups. The Oil Red-O staining of liver samples further confirmed the effects of exercise on reducing lipid deposition in the liver.

### Effects of HIIT and MICT on mitochondrial lipid metabolic enzymatic activity and mitochondrial biogenesis

**Fig. 4:**
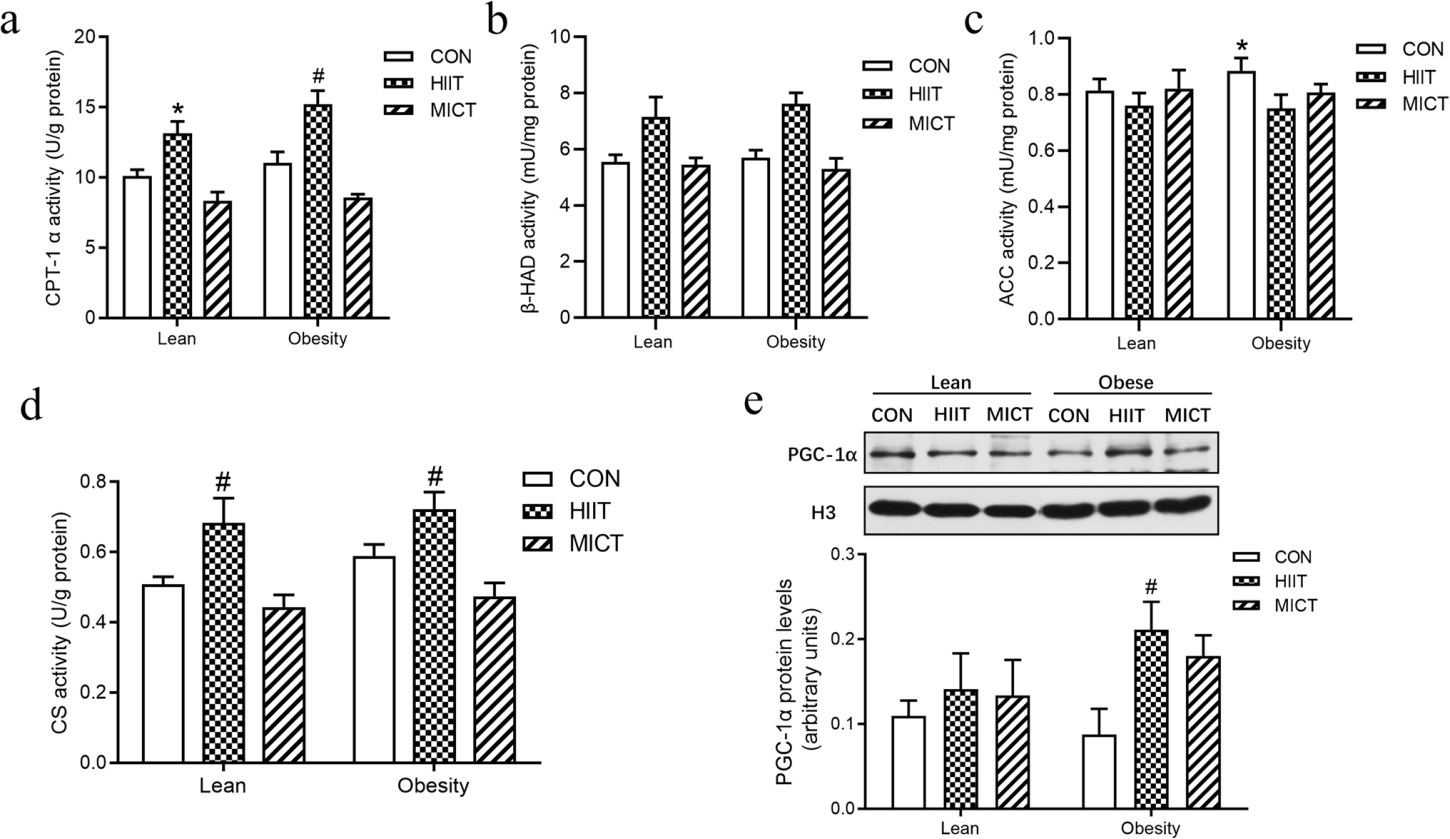
Effects of HIIT and MICT on hepatic mitochondrial-lipid metabolic enzymatic activity. Alterations in mitochondrial carnitine palmitoyltransferase-1 alpha (CPT-1α) (**a**), β-hydroxyacyl-CoA dehydrogenase (β-HAD) (**b**), and acetyl-CoA carboxylase (ACC) activities (**c**). Changes in mitochondrial maximal citrate synthase (CS) activity (**d**). Nuclear PGC-1α (91 kDa) protein content (**e**). LC, lean + quiet control group; LHI, lean + high-intensity interval exercise training group; LMI, lean + moderated intensity continuous training group; OC, obesity + quiet control group; OHI, obesity + high-intensity interval exercise training group; OMI, obesity + moderated intensity continuous training group. Values are presented as the mean ± SD (*n* = 6–8 per group). *P < 0.05 vs LC group (*t*-test or one-way ANOVA), #*P* < 0.05 vs OC group (one-way ANOVA)

To evaluate whether the levels of mitochondrial biogenesis and lipid metabolic enzymatic activities were associated with the observed decreases in liver TG content, we determined that 8-week HIIT significantly increased the expression of peroxisome proliferator–activated receptor gamma coactivator-1 (PGC-1), a key regulator of mitochondrial biogenesis, as well as mitochondrial function, as measured by citrate synthase (CS) enzyme activity. Furthermore, ELISA results showed that the activities of mitochondrial lipid oxidative metabolic enzymes—such as carnitine palmitoyltransferase-1 alpha (CPT-1α) and β-hydroxyacyl-CoA dehydrogenase (β-HAD)—were significantly upregulated in both lean and obese groups following HIIT, but no significant change was observed following the MICT intervention (Fig. 3). We also evaluated the activity of acetyl-CoA carboxylase (ACC), the rate-limiting step of de novo lipogenesis and a regulator of fatty-acid β-oxidation in hepatocytes. Compared with that of the LC group, a significantly higher level of ACC activity was found in the obese control groups (Fig. 4).

### Effects of HIIT and MICT on oxidative stress in liver tissue

**Fig. 5:**
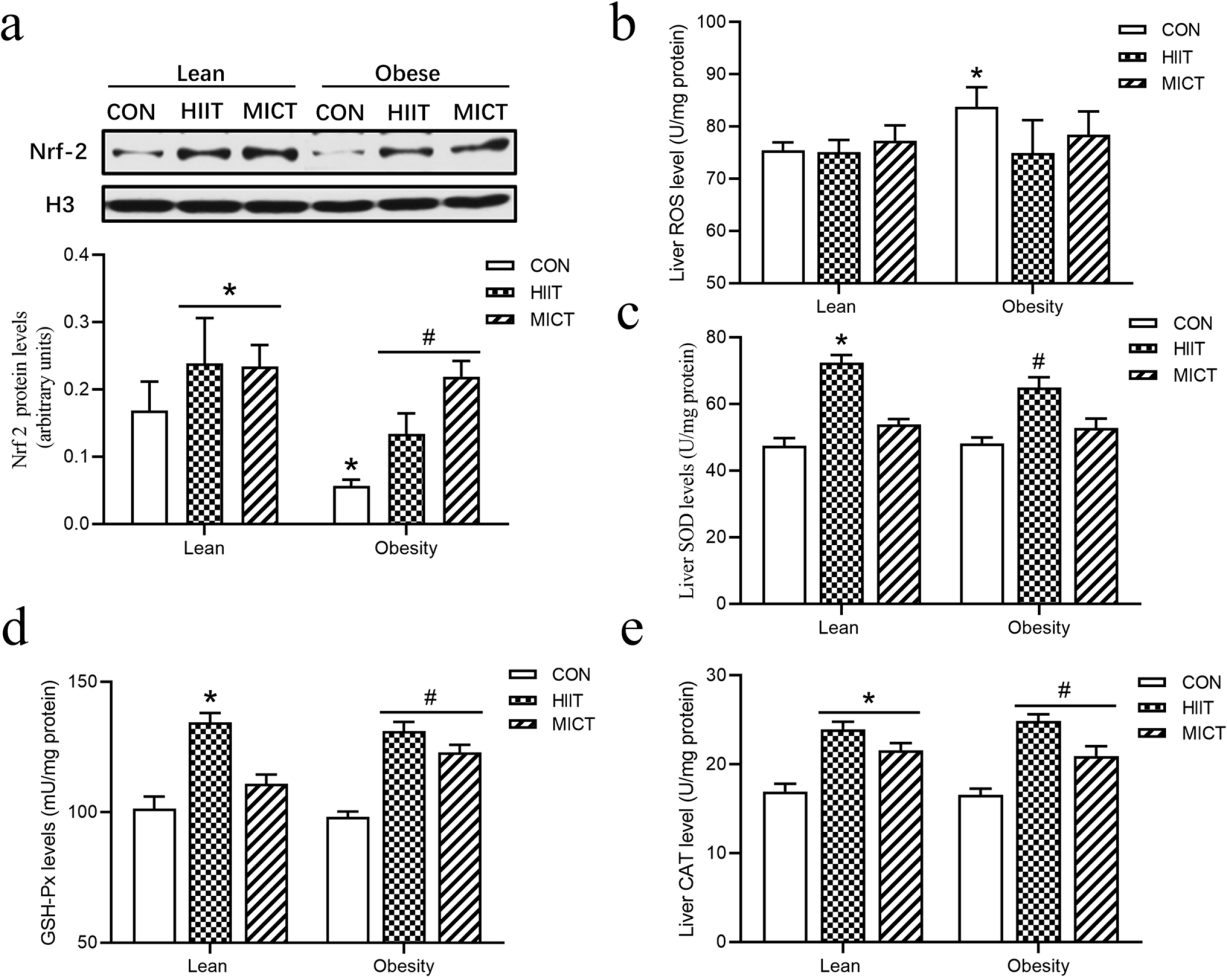
Effects of HIIT and MICT on oxidative stress in liver tissue. Nuclear Nrf 2 (110 kDa) protein content (**a**), liver ROS level (**b**), and the activities of superoxide dismutase (SOD) (**c**), glutathione peroxidase (GSH-Px) (**d**), and catalase (CAT) (**e**). LC, lean + quiet control group; LHI, lean + high-intensity interval exercise training group; LMI, lean + moderated intensity continuous training group; OC, obesity + quiet control group; OHI, obesity + high-intensity interval exercise training group; OMI, obesity + moderated intensity continuous training group. Values are presented as the mean ± SD (*n* = 6–8 per group). *P < 0.05 vs LC group (*t*-test or one-way ANOVA), #*P* < 0.05 vs OC group (one-way ANOVA)

In order to explore the effect of HIIT and MICT on oxidative stress in the liver, ROS level, nuclear factor erythroid 2 (NFE2)–related factor 2 (Nrf-2) expression, and antioxidant enzyme were detected (Fig. 5). There were significantly increased ROS level in obese rats compared with lean control group, and this alteration was attenuated with HIIT and MICT intervention. Reactive oxygen species (ROS) is harmful to our health, and superoxide dismutase (SOD), glutathione peroxidase (GSH-Px), and catalase (CAT) are the major antioxidant enzymes that defend us from effects of ROS. In addition, many of the antioxidant enzymes were regulated by Nrf2 within the cell to regulate redox signaling in the local environment. As the results shown (Fig. 5), there were downregulation in protein expressions of Nrf2 in obese rats compared with lean rats and a significant increase in HIIT and MICT groups compared with control groups in both lean and obese animals. Moreover, we also observed a significant increase in the activity of SOD, GSH-Px, and CAT level with exercise intervention.

### HFD-induced hepatic ER stress is mitigated following HIIT and MICT via the PERK-ATF4-CHOP pathway

**Fig. 6:**
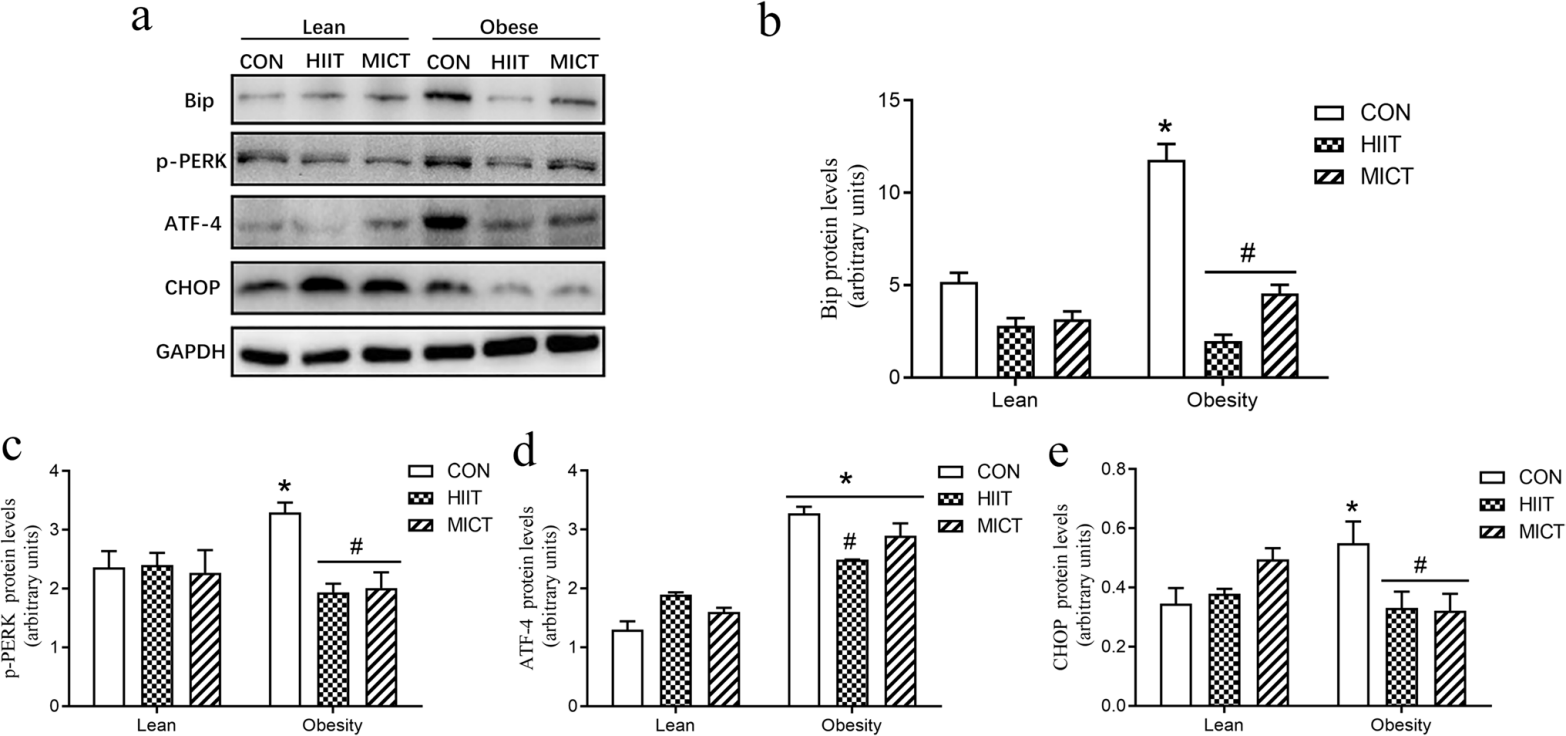
Protein expression levels of components of the PERK-ATF4-CHOP pathway after HIIT and MICT. Immunoblotting of Bip (78 kDa), p-PERK (125 kDa), ATF-4 (49 kDa), CHOP (27 kDa), and GAPDH (36 kDa) in the experimental groups (**a**). Densitometric analysis of Bip (**b**), p-PERK (**c**), ATF-4 (**d**), and CHOP (**e**) proteins in different groups. Values are presented as mean ± SD (*n* = 6–8 per group). *P < 0.05 vs LC group (*t*-test or two-way ANOVA), #*P* < 0.05 vs OC group (one-way ANOVA)

The results of Western blotting revealed that obese rats had higher levels of ER stress–related protein expression than those of lean rats (Fig. 6). Nevertheless, HIIT and CT significantly decreased Bip expression levels in both lean and obese groups. However, HIIT only significantly reduced the expression levels of p-PERK and ATF-4 in obese rats compared with those in the control groups. We also determined the protein expression levels of CHOP, a molecule involved in ER stress–induced apoptosis, that was predominantly expressed in the OC group. Although no significant changes were observed in lean rats, both HIIT and MICT promoted significant downregulation of CHOP expression in obese rats.

## Discussion

In recent years, there has been a growing interest in studying the different impacts of HIIT and MICT exercise modalities on energy expenditure and the metabolism of lipid oxidation in obesity [7, 18, 29, 35]. Here, in an experimental model, we demonstrated HFD-induced hepatic lipid accumulation and enhanced ER stress levels. However, exercise training, particularly HIIT, reversed these changes likely by downregulating PERK-ATF4-CHOP signaling pathways. These findings were consistent with reduced serum lipid levels and inflammatory markers, as well as increased activities of mitochondrial-lipid oxidative enzymes, enhanced antioxidant enzyme activities in liver tissue.

There is robust and growing evidence that HIIT may elicit superior benefits compared to those of MICT across a range of health markers in both healthy and chronically ill populations. Recent meta-analyses have reported that HIIT induces greater improvements in cardiorespiratory fitness than those of MICT in healthy, young, and middle-aged adults [19] , as well as in patients with coronary artery disease and cardio-metabolic disorders [9, 33]. A previous study reported that compared with that of traditional exercise interventions, vigorous physical activity showed a significant preventative effect in the progression of fatty liver to NASH [27]. Modified HIIT of five cycles of high-intensity cycling followed by 3-min recovery periods, three times/week for 12 weeks, demonstrated a reduction in liver fat and improvement in early diastolic filling in 23 NAFLD patients compared with those of standard controls [13]. The present study examined the differential effects of HIIT and MICT on liver fat metabolism in normal and obese mice. We found that compared with those of normal-diet mice, HFD mice developed a stably higher body weight, as well as abnormal serum lipid and inflammatory factor levels. HIIT and MICT showed similar effects on decreased body weight, ameliorating dyslipidemia, and reducing serum inflammatory markers in HFD mice. Furthermore, HIIT demonstrated a decrease in body weight, notably in obese mice; however, CT displayed a greater effect in lean mice.

The liver is an essential organ for lipid metabolism. As a central regulator of lipid homeostasis, the liver is responsible for orchestrating the synthesis of new fatty acids, their export, and subsequent redistribution to other tissues, as well as their utilization as energy substrates. The uptake of fatty acids in the liver from the plasma is uncontrolled and driven by free fatty acid (FFA) plasma levels. If the liver is taking up more fatty acids than it can use in the VLDL formation and excretion, these surplus fatty acids will be stored in the liver in the form of fat droplets. Our present findings showed that following a HFD and lack of exercise, a dyslipidemic phenotype emerged that was characterized by increased TG levels, decreased HDL levels, and a shift in LDL proteins to a more proatherogenic composition (small dense LDL) [28], suggesting that a flux of FFAs to the liver was profoundly increased and likely contributed to increased fat accumulation within the liver. In MICT exercise mode, skeletal muscle consumes more lipids from circulation because of its lower exercise intensity; thus, MICT has stronger effect on changing the systemic lipid levels, such as serum TG or cholesterol than HIIT.

Intrahepatic lipid levels are governed by the balance between lipid acquisition and disposal. The liver acquires lipids through the uptake of circulating fatty acids and via de novo lipogenesis [14]. Numerous studies have shown that exercise training significantly decreases intrahepatic fat content. The underlying mechanism involves β-oxidation and lipogenesis. β-Oxidation has been found to be increased in human NASH [21]. However, structural defects to liver mitochondria have indicated that a compensatory increase in β-oxidation may lead to mitochondrial damage and dysfunction in the long term. Studies in rodents have confirmed that exercise improves liver mitochondrial function and increases palmitate oxidation in freshly excised livers, concomitantly with an increase in hepatic carnitine palmitoylCoA transferase 1 (CPT-1) [4, 17], an enzyme necessary for transport of FA from the cytosol across the mitochondrial membrane. In our present study, we observed an increase in CPT-1 and β-HAD activities with HIIT training in both lean and obese mice, but this adaptation was not found in MICT-trained rats. In MICT exercise mode, skeletal muscle uses more lipids from circulation as its lower exercise intensity; thus, the amount of uptake fatty acids to the liver will be decreased. Although without altering mitochondrial FAO enzyme activities. Conversely, HIIT exercise mode relies more on glycometabolism and less on lipid, which results in more fatty acids left in circulation and higher level of fatty acids uptake to the liver. Therefore, it is easier to see the adaptive changes in mitochondrial FAO enzyme activities under HIIT intervention rather than MICT.

In addition, CS activity has been suggested as a marker of mitochondrial content. Consistent with the enhanced activities of mitochondrial-lipid oxidative metabolic enzymes, data from the present study indicated that there was an increase in the pool of mitochondria from rats in the HIIT-trained group, but no such alterations were found due to MICT training. Moreover, the levels of peroxisome proliferator–activated receptor-gamma coactivator-1 alpha (PGC-1α), a key player in the regulation of mitochondria biogenesis, were significantly higher in obese exercised rats in comparison with those of obese sedentary rats. Moreover, in lipogenesis, acetyl-coA derived from the Krebs cycle gets converted into long-chain fatty acids facilitated by a number of enzymes, such as acetyl-CoA carboxylase (ACC) and fatty acid synthase (FAS). Animal models have shown that exercise results in increased levels of phosphorylated AMPK and ACC in livers and, therefore, inactivation of ACC enzymatic activity [4, 34]. In the current study, we observed that the significant increase of ACC activity in obese mice was attenuated in obese trained rats, which might represent an adaptive response to increased lipolysis observed in the exercise groups.

HFDs are known to contribute to the development of obesity and related comorbidities, including NAFLD. Abnormal lipid accumulation is often associated with insulin resistance in steatotic livers and coincides with oxidative stress and perturbed ER proteostasis in hepatocytes. Accumulating evidence indicates that the liver’s role in regulating the immune response is linked to ER stress and inflammation. It is known that certain inflammatory mediators—such as pro-inflammatory cytokines including IL-1β, IL-6, and TNF-α—induce ER stress [22, 24, 32]. Our present study demonstrated that both HIIT and MICT interventions dramatically suppressed elevated levels of serum inflammatory markers (i.e., MCP-1, TNF-α, and IL-1β) compared to those in obese sedentary mice. We also examined hepatic oxidative stress [5]. The increased influx of FFAs induces overloaded β-oxidation and subsequent mitochondrial dysfunction in obese rats. Literature has shown that FAO-originated ROS are a major source of ROS and a crucial player in oxidative stress during hepatic FFA overload [3]. Mitochondrial dysfunction and endoplasmic reticulum (ER) stress are the primary mechanisms that account for increased generation of reactive oxygen species (ROS) [31]. Therefore, hepatic mitochondrial-lipid metabolic enzymatic activity and mitochondrial function are related to FAO capacity, lower FAO capacity could induce higher oxidative stress, and increased ROS generation induces ER stress changes [15]. We observed in our study that there were increase in ROS level and decrease in Nrf2 expression in obese sedentary rats; HIIT and MICT significantly reduce oxidative stress in liver tissue by upregulating Nrf2 expression and antioxidant enzyme activities. These results implied that overloaded β-oxidation in obese rats could induce the oxidative stress in liver tissue, while exercise intervention protects the liver against oxidative stress.

To further investigate whether this HFD-induced increased inflammation and oxidative stress contributed to ER stress, we examined the expression of immunoglobulin heavy chain binding protein (BiP), also referred to as 78-kDa glucose-regulated protein (GRP78), which is a pivotal ER chaperone that modulates the unfolded protein response under ER stress. The results showed a positive association between increased inflammatory markers and Bip expression, corroborating the link between HFD-induced inflammation and ER stress. Furthermore, various components of ER stress play roles in regulation of lipid metabolism, and the PERK-eIF2α-ATF4 pathway has been identified as a critical component required for regulating lipogenesis and hepatic steatosis [1]. In the present study, the expression levels of GRP78, ATF4, and CHOP were upregulated, and the phosphorylation of PERK was increased, in the livers of mice with HFD-induced obesity, which were reduced by both HIIT and MICT training for 8 weeks. HIIT or MICT intervention effectively reduced ER stress in obese rats, but not in lean rats. Moreover, activation of ATF4, which is induced by PERK-mediated phosphorylation of eIF2α, is thought to play a dominant role in the induction of CHOP in response to ER stress [15]. In line with the ER stress pathway, the transcription factor CCAAT-enhancer-binding protein homologous protein (CHOP)—which was first reported as a molecule involved in ER stress–induced apoptosis—also showed similar changes with diet or exercise interventions in the present study. Specifically, CHOP expression was markedly increased in response to diet-induced ER stress, but was significantly decreased under HIIT or MICT intervention. Therefore, these results indicate that unresolved ER stress under obese conditions induces dysregulation of hepatic lipid metabolism. However, HIIT and MICT effectively attenuate this excessive ER stress and ameliorate HFD-induced hepatic lipid accumulation.

In conclusion, we found that both HIIT and MICT were effective in controlling body weight gain and inflammatory levels in obese rats and alleviated ER stress via modulating PERK-ATF4-CHOP signaling pathways in the liver, reverting HFD-induced pathoanatomical features of the liver [34]. However, HIIT was more efficient than was MICT in upregulating activities of mitochondrial-lipid oxidative metabolic enzymes. This suggests that adaptation of oxidative metabolism of mitochondrial responses exhibits differential mechanisms via HIIT and MICT, which could result in hepatic lipid metabolism via different pathways. Taken together, our findings suggest that both HIIT and MICT may represent effective interventions for correcting deficiencies in HFD mice by suppressing lipid deposition in the liver.

Unfortunately, the present study has limitations; especially, the underline mechanism of exercise on ER stress remains to be better understood. Although the current data demonstrated that exercise alleviates lipid deposition in hepatocytes accompanied by decreased endoplasmic reticulum stress levels (PERK-ATF4-CHOP signaling pathways), it is not yet clear whether exercise training improves ER stress by reducing the oxidative stress levels in hepatocytes or by a direct action on endoplasmic reticulum stress. In addition, currently, we are unable, in our laboratory, to measure the rate of mitochondrial fatty acid oxidation as ^14^CO_2_ generation from ^14^C palmitate; future studies will try to use more accurate methods. Overall, this study provides evidence that different exercise modes reduce endoplasmic reticulum stress and improve liver fat deposition induced by high-fat diet, and the molecular mechanism of which needs to be further studied.
